# Altered ureido protein modification profiles in seminal plasma extracellular vesicles of non-normozoospermic men

**DOI:** 10.3389/fendo.2023.1113824

**Published:** 2023-03-22

**Authors:** Rosa Roy, Cristina Lorca, María Mulet, José Antonio Sánchez Milán, Alejandro Baratas, Moisés de la Casa, Carme Espinet, Aida Serra, Xavier Gallart-Palau

**Affiliations:** ^1^ Department of Biology, Genetics Unit, Universidad Autónoma de Madrid, Cantoblanco, Madrid, Spain; ^2^ Biomedical Research Institute of Lleida (IRBLLEIDA), +Pec Proteomics Research Group (+PPRG), Neuroscience Area, University Hospital Arnau de Vilanova (HUAV), Lleida, Spain; ^3^ IMDEA-Food Research Institute, Campus of International Excellence UAM+CSIC, Old Cantoblanco Hospital, Madrid, Spain; ^4^ GINEFIV, Assisted Reproduction Centre, Madrid, Spain; ^5^ Department of Medical Basic Sciences, University of Lleida (UdL), Lleida, Spain; ^6^ Department of Psychology, University of Lleida (UdL), Lleida, Spain

**Keywords:** seminal plasma, citrullination, deimination, carbamylation, citrulline, homocitrulline, ureido protein modifications, exosomes

## Abstract

**Introduction:**

Extracellular vesicles (EVs) have been recognized as key players in numerous physiological functions. These vesicles alter their compositions attuned to the health and disease states of the organism. In men, significant changes in the proteomic composition(s) of seminal plasma EVs (sEVs) have already been found to be related to infertility.

**Methods:**

Methods: In this study, we analyze the posttranslational configuration of sEV proteomes from normozoospermic (NZ) men and non-normozoospermic (non-NZ) men diagnosed with teratozoospermia and/or asthenozoospermia by unbiased, discovery-driven proteomics and advanced bioinformatics, specifically focusing on citrulline (Cit) and homocitrulline (hCit) posttranscriptional residues, both considered product of ureido protein modifications.

**Results and discussion:**

Significant increase in the proteome-wide cumulative presence of hCit together with downregulation of Cit in specific proteins related to decisive molecular functions have been encountered in sEVs of non-NZ subjects. These findings identify novel culprits with a higher chance of affecting fundamental aspects of sperm functional quality and define potential specific diagnostic and prognostic non-invasive markers for male infertility.

## Introduction

Infertility is estimated to affect over 70 million people worldwide, and men’s contributions to couple infertility are estimated to involve nearly 50% of the cases (1). Of note, 30% of these men-linked infertility contributions involve idiopathic sperm abnormalities (1). Although research on spermatozoa has traditionally centered the research efforts performed to understand male infertility, the weightiness of seminal plasma (SP) within the intricate process of fertilization has also been accounted for (2). SP is a rich fluid, secreted by the epididymis and accessory sexual glands in the male reproductive tract, that regulates semen viscosity, spermatozoa transit, immunologic uterine tolerance, and fertilizing capacity (3). These regulatory actions were initially attributed to the free SP circulating molecules; however, parallel findings indicated that SP contains a large number of extracellular vesicles (EVs), though the role(s) of these vesicles have remained poorly understood for a long time (4). Recent findings have demonstrated that the molecules associated with these vesicles significantly contribute to some essential structural and functional roles initially attributed to SP (5). Thus, implementing further research actions aimed to elucidate the role(s) and characteristics of seminal plasma EVs (sEVs) is most necessary.

sEVs, also known as prostasomes, are tiny bi-layered vesicles that commonly range in diameter from 30 nm to 1 μm, with exosomes (<250 nm) and microvesicles (>250 nm) being the main components of the distribution (6, 7). The membrane of these vesicles is structurally formed by lipids and multidomain proteins, both of which possess outstanding signaling abilities (8). Furthermore, EVs and prostasomes are known to contain proteins, lipids, and genetic material as cargo, ready to be taken up by the target recipient (9). It has been widely shown that these circulating vesicles alter their biogenesis and molecular composition(s), which are attuned to the rhythms of human health and disease (10–12), and male infertility does not lack the ability to exert that influence (13). Although changes affecting sEV proteomes at the protein level have been already analyzed in healthy individuals (14) and male infertility conditions (15), the posttranslational biochemical profile(s) of sEV proteomes in this disorder remain mostly unexplored. Although, recent advances, aimed at characterizing posttranslational modifications in prostasomes, have identified that specific glycan modifications, affecting the proteome of these vesicles, allow distinction between normozoospermic and oligozoospermic men profiles (16).

Posttranslational modifications (PTMs) have the ability to regulate the function, stability, interaction, localization, and turnover of translated proteins (17). These functional and dynamic decorations tend to present standard molecular patterns within certain eukaryotic proteomes, though their profiles are highly susceptible to reflecting the effects of disordered conditions (18). Furthermore, PTMs not only exert regulatory functions over the proteome of eukaryotic cells, but these modifications also affect proteins implicated in intercellular communication, such as those in EVs (19). Indeed, the identification and characterization of disorder-related PTMs in bodily circulating EVs have been postulated as a next-generation paradigm for the identification of more specific molecular biological markers (20). Although certain PTMs, such as phosphorylation and glycosylation, have regained an outstanding amount of interest within the scientific community, others, such as citrullination and carbamylation, have remained further in the shade (21, 22). This is partially due to the fact that progress on their biochemical characterization in health and disease conditions cannot be properly achieved, as with the most popular PTMs, by the use of classic biochemical methods (e.g., using specific antibodies), but it requires implementation of advanced systems biology technologies (22–25).

In fact, non-coded citrulline residues (Cit) become a product of enzymatic citrullination of Arg by peptidylarginine deiminases (PADs) and are almost biochemically indistinguishable from homocitrulline (hCit), which in turn is the product of Lys carbamylation through spontaneous reaction with isocyanic acid (25). Cit and hCit are considered ureido protein modifications (uPMs) as both share an identical ureido group and only differ by one carbon in the side chain (22, 23). Specific biochemical characterization of uPMs at the single protein or at proteome-wide levels can only be achieved by the use of advanced mass spectrometry technologies (22, 23, 26).

In a related vein, uPMs have been proposed as protein decorations strongly implicated in the regulation of fertility in mammals (27). However, to the best of our knowledge, any potential role(s) of uPMs in human sEVs are yet to be defined. Thus, in this work, we focus on analyzing, by unbiased discovery-driven proteomics, uPMs in sEVs of subjects diagnosed with non-normozoospermic (non-NZ) disorder. The findings obtained indicate that although significant differences could be identified in the cumulative hCit occurrence in sEV proteomes, protein-specific uPM alterations in sEVs of non-NZ men were only attributable to Cit residues.

## Materials and methods

### Clinical samples

Human semen samples (*n* = 26) were generously provided by the fertility clinic GINEFIV (Madrid, Spain). These clinical samples were donated by healthy men with NZ sperm profile(s) (NZ; *n* = 12) and healthy men diagnosed with teratozoospermia and/or asthenozoospermia (non-NZ; *n* = 14). All semen samples were obtained by masturbation after 4 days of sexual abstinence and were allowed to liquefy before experimental diagnosis for 30–60 min at 37°C in a CO_2_ incubator (5% CO_2_ in air at 95% relative humidity). All non-NZ subjects showed abnormal reproductive clinical history (no history of previous partner pregnancy or clinical history of being involved in pregnancy loss), abnormal semen analysis, and a clinical diagnostic for male infertility at the time of inclusion in the study, based on the reported clinical guidelines for the diagnosis of male infertility (28). Clinical samples were cryopreserved at −150°C until further analyses. The use of human samples was performed in strict accordance with the Declaration of Helsinki. The following detailed experimental procedures were performed according to institutional guidelines and were previously approved by the Ethics Committee of the Autonomous University of Madrid (UAM) with reference: CEI 60-1058.

### Isolation of seminal plasma extracellular vesicles

Semen samples were thawed till liquification and centrifuged at 500×*g* for 10 min to remove spermatozoa and cell debris. Processed samples were then visually inspected under the microscope to confirm the absence of spermatozoa and subsequently stored at −80°C prior to the isolation of sEVs.

Isolation of sEVs was performed by sequential centrifugation as follows: thawed SP samples were centrifuged at 3,000×*g* for 20 min, and the supernatant was centrifuged at 12,000×*g* for 20 min. sEVs were then pelleted down by ultracentrifugation at 100,000×*g* for 70 min using a Beckman 70Ti rotor. Subsequently, the pellet containing the sEVs was further washed by mixing it with 20 ml of PBS, and sEVs were pelleted down again at 100,000×*g* for 70 min. sEVs were resuspended in 200 µl of 1× PBS and stored at −80°C before further processing.

### Characterization of sEVs by nanoparticle tracking analysis

Preparations of sEVs were analyzed by nanoparticle tracking analysis (NTA), as we previously indicated (8). The NTA experiments were performed in triplicate. Briefly, samples were diluted to 1:1,000 in 1× PBS and analyzed by using the NanoSight System (NanoSight, Malvern Instruments, UK), equipped with a 405-nm laser. No restrictions were applied to the imaged fields.

### Ultrastructural characterization of sEVs by transfer electron microscopy

Isolated sEVs from representative samples (two NZ and two non-NZ) were mounted on Cu-Formvar-carbon grids. After 20 min at RT, grids were washed with distilled water, and sEV samples were fixed with 1% glutaraldehyde for 5 min in PBS. sEV samples were then stained with uranyl oxalate for 5 min. Subsequently, samples were embedded in methyl-cellulose-uranyl-oxalate and dried for permanent preservation. Electron micrographs were collected using a Jeol Jem 1010 electron microscope at 80kV.

The obtained TEM micrographs were scale calibrated and bi-leveled in ImageJ (National Institutes of Health (NIH), USA). Particle diameter sizes, for all observed particles in each micrograph, were independently analyzed and quantified without constraints.

### In-gel tryptic digestion and isobaric peptide labeling of sEV proteomes

Protein quantitation of sEV proteomes was performed by a bicinchoninic acid assay using a BCA kit (Thermo Scientific, Waltham, MA, USA) following the manufacturer’s instructions. sEV samples were then combined to obtain three NZ and four non-NZ pools with an average protein concentration of 100 µg, as previously detailed (15). sEV samples were subjected to in-gel digestion as previously described (29), with minor modifications. Briefly, sEVs were suspended in 50 µl of sample buffer and placed into a 1.2-cm-wide well of SDS-PAGE gel (0.75 mm thick, 4% stacking, and 10% resolving). The run was stopped when the sample front reached 3 mm into the resolving gel; hence, the whole proteome was concentrated in the resolving gel interface. Coomassie staining was used to visualize the unseparated protein bands. A gel containing the protein band was cut into cubes of approximately 2 × 2 mm. The gel pieces were destained using acetonitrile:water (1:1). Protein disulfide bonds were reduced with 10 mM DTT for 1 h at 56°C and cysteines were alkylated with 50 mM iodoacetamide for 1 h at room temperature in darkness protected from the light. After, gel pieces were dehydrated using acetonitrile (ACN), and 50 mM ammonium bicarbonate at pH 8.8 with 60 ng/µL sequencing grade trypsin (Promega, Madison, WI, USA) was added to the dried gel pieces. Samples were then placed on ice for 2 h to ensure proper hydration. Trypsin digestion was performed at 37°C for 12 h. Digestion was then quenched by acidification with 1% trifluoracetic acid. Digested peptides were extracted by using 50% ACN and 5% acetic acid in at least three rounds under ultrasound sonication. Supernatants containing the digested sEV proteomes were collected and dried in a vacuum concentrator.

sEV proteomes were then labeled with an 8-plex isobaric tags using the iTRAQ 8plex Multiplex kit (Applied Biosystems, Foster City, CA) according to the manufacturer’s protocol. iTRAQ isobaric groups were distributed as follows: 113 healthy control (NZ1), 114 healthy control (NZ2), 119 healthy control (NZ3), 115 non-normozoospermic (non-NZ1), 116 non-normozoospermic (non-NZ2), 117 non-normozoospermic (non-NZ3), 118 non-normozoospermic (non-NZ4), and 121 non-normozoospermic (non-NZ5). Labeled peptides were combined and desalted using an OASIS HLB desalting cartridge (Waters Corporation, Milford, MA, USA), and the eluates were dried to completion in a vacuum concentrator, prior to being simplified into three cartridge-base separated fractions.

### Reversed-phase liquid chromatography-tandem mass spectrometry of sEV proteomes

LC-MS/MS analysis of the labeled sEV proteomes was performed using an Easy-nLC II system coupled to an ion trap LTQ-Orbitrap Velos Pro hybrid mass spectrometer (Thermo Scientific, Waltham, MA, USA), as previously detailed (15, 30). Briefly, dried peptides were resuspended in 10 µl of 0.1% formic acid and reversed-phase chromatographic separation was carried out using a 0.1 mm × 20 mm C18 RP precolumn (Phonomenex, Torrance, CA, USA) and a 0.075 mm × 250 mm C18 RP column (Phonomenex, Torrance, CA, USA) operating at 300 nl/min. The separation of peptides was conducted in a 240-min gradient as follows (solvent A: 0.1% formic acid in water; solvent B: 0.1% formic acid, 80% acetonitrile in water): from 5% to 25% solvent B in 180 min followed by a gradient from 25% to 40% solvent B over 240 min. Ionization of peptides was performed using a Nano-bore emitter Stainless Steel ID 30 µm (Phonomenex, Torrance, CA, USA) interface. Data acquisition was conducted for the top 20 ions with an Orbitrap MS scan at a resolution of 30,000, followed by 20 high-energy collision dissociation (HCD) MS/MS scans performed in the Orbitrap at a resolution of 7,500. The minimum MS signal for triggering MS/MS was 500. The lock mass option was enabled for both MS and MS/MS modes, and polydimethylcyclosiloxane ions (protonated (Si(CH3)2O))6; *m/z* 445.120025) were used for internal recalibration. Peptide detection was performed in survey scans from 400 to 1,600 amu (1 µscan) with an isolation width of 2 u (mass-to-charge ratio units), a normalized collision energy of 40% for HCD fragmentation, and a 30-s dynamic exclusion window. Unknown or +1 charge state precursors were discarded.

### Bioinformatics and data analysis

Analysis of isobaric iTRAQ labeling raw data was carried out using the specialized bioinformatics suite software PEAKS Studio Xpro version 10.6 (Bioinformatics Solutions Inc., Waterloo, Ontario, Canada). The UniProt human database lastly modified on 5 November 2019 with 74,788 sequences used for searching. For protein identifications, carbamidomethyl of Cys was set as a fixed modification. PTM algorithm available in PEAKS Studio Xpro software was used for the identification of protein PTMs. Tolerances of 10 ppm for precursor ions and 0.05 Da for MS/MS fragment ions were used. FDR < 1% was established for protein identification in all samples (31) and trypsin was set as a proteolytic enzyme. Data were exported to Microsoft Excel CSV files and in-house-generated macros were used for protein quantification analyses. Data were analyzed considering a significance threshold of *p* < 0.05 and were reported as mean ± standard deviation (SD), if not otherwise specified. Identification of exosome and microvesicle markers was performed by comparison with the top 100 EV markers curated in the specialized databases Exocarta (32) and Vesiclepedia (33). Sequence alignment was performed using the BLOSUM62 substitution matrix in Jalview version 2.11.2.3 considering the Cit residues ± 10 residues. Functional motif analysis was performed using the Interpro protein families and domains database (34). GraphPad Prism version 8.4.3 was used for parametric and nonparametric statistical analyses and for creating and rendering data plots. The potential interaction of age with each of the clinical variables that showed significant differences in NZ subjects was assessed by Pearson’s (*p* < 0.05). Figures were assembled in the final version using the Illustrator 2020 software (Adobe, San José, CA, USA).

## Results

### Identification of sperm parameters linked to male infertility in non-NZ donors

**Figure 1:**
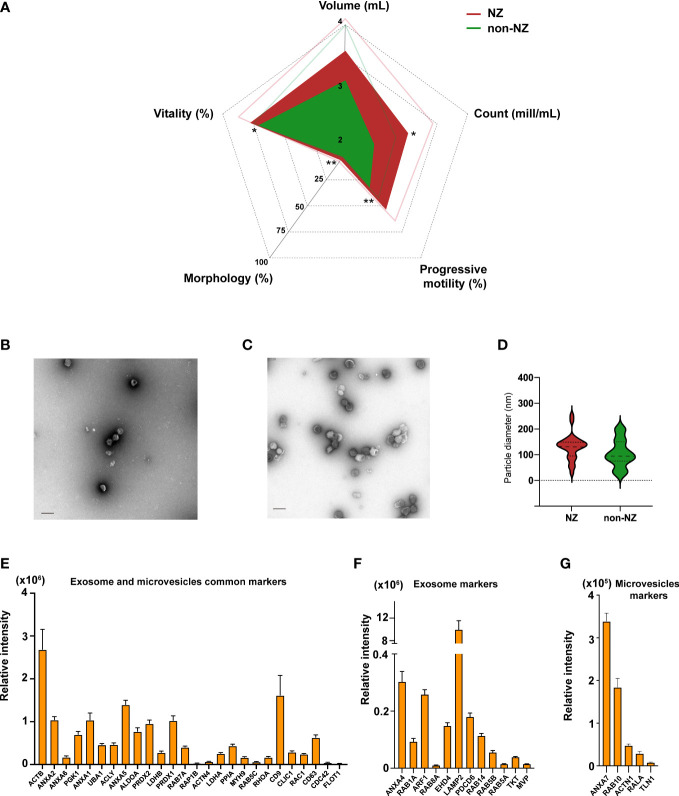
Analyses of clinical sperm parameters in normozoospermic (NZ) and non-normozoospermic (non-NZ) men and physicochemical characterization of sEVs. **(A)** Spider web chart displaying the average sperm parameters of NZ and non-NZ subjects. Thin lines represent the standard deviation. Significant differences between groups were assessed by Student’s *t*-test (significance established at *p* ≤ 0.05), and sperm progressive motility was the most significantly affected clinical sign of non-NZ men followed by count and vitality, and morphology. Ultrastructural characterization of sEVs from **(B)** non-NZ men and **(C)** NZ men. **(D)** Violin graph depicting vesicle size quantification from the obtained micrographs of sEVs of non-NZ men and NZ. No statistically significant differences in particle size were observed between the analyzed groups (*p* < 0.05). **(E)** Bar chart indicating the average relative intensity detected by LC-MS/MS of EV markers commonly curated in the specialized databases Exocarta and Vesiclepedia identified in sEVs. **(F)** Bar chart indicating the average relative intensity detected by LC-MS/MS of specific exosome markers curated in the specialized database Exocarta identified in sEVs. **(G)** Bar chart indicating the average relative intensity detected by LC-MS/MS of specific microvesicle markers curated in the specialized database Vesiclepedia identified in sEVs. ^*^ *p* ≤ 0.05 and ^**^ *p* < 0.01—levels of significant differences. Scale bars in transfer electron microscopy micrographs represent 200 nm.

To define whether non-NZ donors present any significant decay in sperm parameters linked to male infertility (35), we comparatively analyzed the available clinical data of the donors included in this study. Analysis of these clinical data revealed that non-NZ donors showed collective impairment of the following sperm parameters compared to NZ donors: vitality, morphology, progressive motility, and count (**Figure 1A**). We then analyzed whether other relevant clinical signs, such as diagnosed atherogenicity and urology history, or relevant lifestyle parameters, such as smoking, alcohol consumption, and toxicology, could have any effect on the encountered non-NZ infertility profiles; no significant effect for these variables was encountered in these analyses (**Supplementary Table S1**). We also analyzed any potential effect of the variable age on the clinical signs that showed significant differences between NZ and non-NZ men, and no significant effect of age over the referred significantly modulated variables was identified (**Supplementary Table S2**). Further details on these donors’ clinical data can be found in **Supplementary Table S1**.

### Ultrastructural and molecular characterization of non-NZ sEVs

NTA and ultrastructural characterization of sEVs were then performed to define the concentration, predominant morphology, average diameter, and potential contamination of the isolates (36). A positive and significant correlation between particle and protein concentration was observed (Spearman’s Rho = 0.337; *p* = 0.051), indicating that sEV abundance was nicely linked to protein abundance in these preparations and that no relevant presence of residual contaminating proteins was present (**Supplementary Figure S1**; **Supplementary Table S3**). Morphologically, these vesicles display a well-rounded structure delimited by a singly formed bi-layer membrane, as shown in the representative micrographs included in **Figures 1B, C**. Furthermore, the observed average particle size was <130 nm (**Figure 1D** **),** and no relevant contamination attributable to non-EVs particles was observed. Collectively, our ultrastructural characterization indicated that these vesicle preparations should be predominantly categorized as exosomes, finding it highly consistent with our own and other colleagues’ previous reports (14, 15).

Molecular characterization of sEVs, achieved by discovery-driven shotgun proteomics (**Figures 1E–G**), demonstrated that these vesicles present an abundant array of common EV markers, including the relevant microvesicle- and exosome-linked proteins FLOT1, CD63, CD9, and ANXA2, as shown in **Figure 1E**. Further analysis demonstrated that the presence of specific microvesicle markers, based on data curated in EVpedia (37), was lower than the presence of specific exosome markers, based on data curated in Exocarta (38) (**Figures 1F, G** **)**, reinforcing the conclusion previously reached through ultrastructural characterization of sEVs.

### uPM proteome profiles in non-NZ sEVs

**Figure 2:**
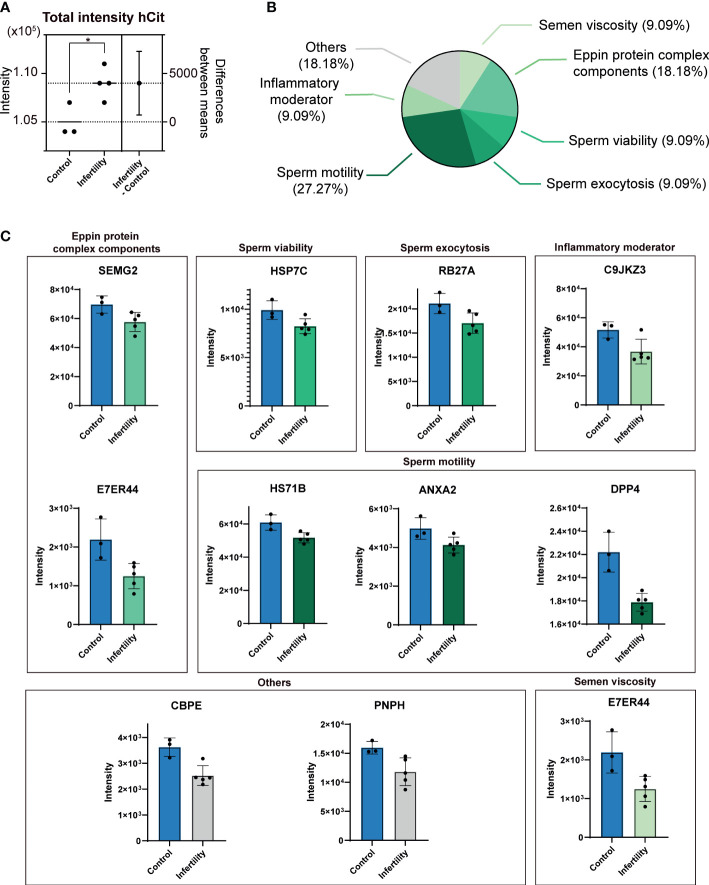
Differential presence of ureido protein modifications (uPMs) in sEV proteomes of non-normozoospermic (non-NZ) men compared to healthy controls (NZ). **(A)** The total cumulative intensity of homocitrullinated (hCit) proteins in sEV proteomes detected in non-NZ and NZ subjects. The intensity was calculated as the sum of spectral counts of all hCit-modified peptides identified. The difference between non-NZ and NZ (nonNZ-NZ) is displayed using the right *y*-axis. **(B)** Functional categorization of Cit proteins in sEV proteomes significantly modulated in non-NZ subjects compared to NZ. **(C)** Relative quantification of significantly modulated citrullinated (Cit) proteins in sEV proteomes from non-NZ and NZ. *p ≤ 0.05, significant differences. Significant differences between groups were assessed by the Student’s t-test.

Subsequently, to investigate specific molecular profiles in these vesicles and to define whether uPMs alter their global proteome-wide occurrence in sEVs linked to non-NZ profiles, we focused on investigating the cumulative presence of uPM-modified proteins in non-NZ men and control subjects (**Supplementary Datasets S1, S2**). Strikingly, we only observed a significant increase in the total levels of hCit affecting sEV proteomes in non-NZ men (**Figure 2A**), while no significant differences were observed regarding the identification of cumulative Cit in these diseased proteomes (**Supplementary Figure S2**). Cumulative hCit in non-NZ samples showed a significant negative correlation with the clinical signs of sperm morphology and progressive motility (**Table 1**), and no significant interaction with age was observed (**Table 1**).

**Table 1:** Correlation analysis between relevant demographic and clinical signs and the hCit cumulative signal in non-NZ samples.

Correlation	Pearson *r*	Significance (*p*)
hCit		
Morphology	−0.5892	0.0266^*^
Motility	−0.6286	0.016^*^
Age	0.3269	0.2713

^*^p < 0.05—significant correlation between variables assessed by Pearson.

Further in-depth scrutiny, at the protein level, indicated that specific differences could not be identified between non-NZ and control subjects regarding the presence of hCit in sEV proteomes. However, significant differences were found between these analyzed groups regarding the occurrence of specific protein Cit, as shown in **Figures 2A, B**. We then performed functional categorization of these significant abnormally citrullinated proteins in sEVs based on previous reports (39–42) and encountered that nearly 30% of these molecules involve in sperm motility, ~20% are components of the Eppin protein complex, and 36% are equally distributed (9% each) in the categories inflammatory moderators, sperm viability, sperm exocytosis, and semen viscosity (**Figure 2B**). The proteins within these categories organized by their SP molecular function and their respective Cit levels in non-NZ and control subjects are shown in **Figure 2C**. Of note, the protein dipeptidyl peptidase-4 (DPP-IV) was the candidate that showed the major significant mismatch in terms of downregulation of its Cit levels between non-NZ and controls, as shown in **Figure 2C**. Other relevant proteins significantly downregulated in their citrullination levels in sEVs of non-NZ subjects include semenogelin-2 (SEMG2), heat shock cognate 71 (HSP7C), Ras oncogene 27A (RAB27A), Annexin 2 (ANXA2), transglutaminase 4 (TGM4), lactotransferrin (LTF), and the carboxypeptidase E (CBPE) (**Figure 2C**).

### Protein uPM stoichiometry in non-NZ sEVs

As previously indicated, we then investigated whether we could identify any significant mismatch between the modified and unmodified portions of the significantly altered citrullinated proteins in non-NZ sEVs. These stoichiometric analyses revealed that a basal level of Cit affecting sEV proteomes involves >25% of the total protein, as detailed in **Table 2**. However, we also strikingly found that the unmodified fraction of the protein was significantly higher in all these sEV molecules in non-NZ men compared to NZ subjects with the exception of the protein RAB27A, which presented a higher portion of its Cit-modified counterpart in non-NZ subjects (**Table 2**).

**Table 2:** Stoichiometric analysis of protein citrullination in sEV proteomes of non-normozoospermic (non-NZ) men compared to normozoospermic (NZ) healthy controls.

Gene symbol	Protein description	Modified residue^a^	% Citrullination^b^		*p* value^c^
			Controls	Patients	
SEMG2	Semenogelin 2	R245	31.75	29.00 ↓^d^	<0.0001
DPP-IV	Dipeptidyl peptidase-4	R611	26.6	23.3 ↓	<0.0001
HSP7C	Heat shock cognate 71 kDa protein	R155	40.7	38.3 ↓	<0.0001
TGM4	Transglutaminase 4	R393	76.7	76.1 ↓	<0.0001
ANXA2	Annexin A2	R196	24.4	23.4 ↓	<0.0001
CBPE	Calcium-binding protein E	R374	52.8	47.7 ↓	<0.0001
C9JKZ3	Transmembrane protease serine 2	R409	17.2	14.6 ↓	<0.0001
RAB27A	Member RAS oncogene family	R80	30.1	31.3 ↑	<0.0001

Only citrullinated (Cit) proteins significantly modified in sEVs of non-NZ subjects compared to NZ are included (p < 0.05 assessed by the Student’s t-test).

a The position of the Cit residue in the protein sequence.

b Percentage of Cit protein compared to unmodified protein based on stoichiometry calculated from intensities of the modified and unmodified Cit peptide detected by mass spectrometry.

c Level of significance obtained from the Xi^2^ analysis.

d Arrows indicate increased (↑) or decreased (↓) stoichiometry compared to controls.

### Functional *in silico* analysis of protein Cit in sEVs of non-NZ men

**Figure 3:**
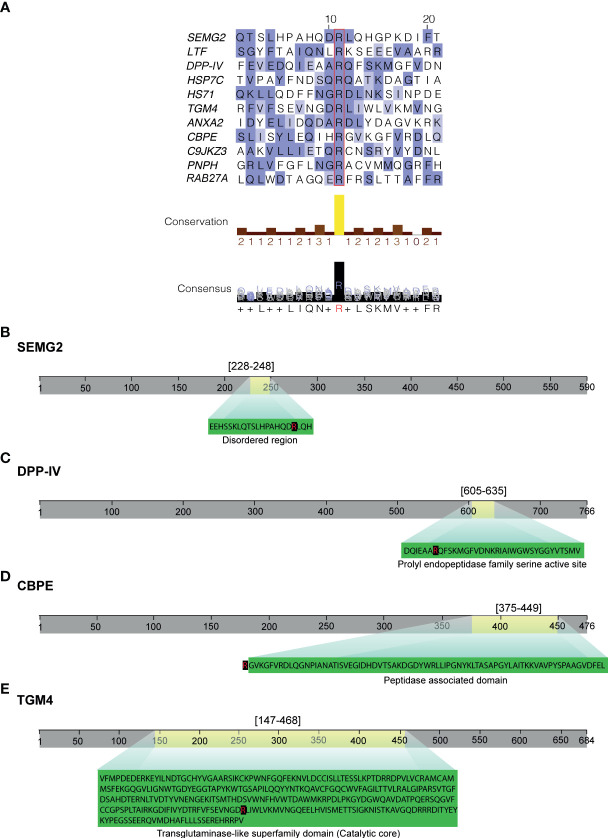
Functional bioinformatics of citrullinated (Cit) proteins in sEV proteomes of non-normozoospermic (non-NZ) men compared to healthy controls (NZ). Only Cit proteins that were significantly modulated in non-NZ compared to NZ (p ≤ 0.05) were considered for these analyses. **(A)** Multiple sequence alignment (Blosum 62) of Cit proteins in sEV proteomes significantly modulated in non-NZ compared to NZ, performed with Jalview. Sequence alignment was performed with partial sequences that formed Cit Arg (squared in red) ± 10 residues. Conserved residues are highlighted in blue shades (mid blue: >60% agreement, light blue: >40% agreement). The amino acid position numbers shown at the top of the sequences correspond to the relative position in the aligned polypeptide. The conservation histogram displayed below the sequence alignment graphic reflects the conservation of physicochemical properties. The yellow column indicates the absolute conservation of Cit Arg (score 11), and less-conserved positions are shown in brown with a decreasing score. The consensus histogram shows the percentage of modal residue per position and includes the conserved sequence logo. “+” indicates non-conserved residues. **(B–E)** Predicting domain analysis performed using the Interpro database to define potential (dis)functional localizations of Cit Arg. Only proteins with relevant colocalizations between Cit Arg and predicted domains are displayed. Grey lines represent the entire sequence length of the protein. Light yellow areas represent the localization of the predicted domains within the entire protein sequence. Green magnifications contain the predicted domain sequence, and the Cit Arg is indicated in red and shaded in black.

Finally, to elucidate whether there may exist a consensus sequence for aberrant Cit in certain proteins of sEVs in non-NZ subjects, similarities between the surrounding area of Cit-modified Arg were *in silico* computed using BLOSUM62 substitution matrix (**Figure 3A**). Sequence alignment results indicated little consensus sequence (< 60% agreement) surrounding Cit residues in the analyzed proteins, with the exception of LTF and CBPE, for which no consensus at all was identified (**Figure 3A**). Subsequently, the potential implications of the aberrant Cit identified in sEV proteomes in non-NZ, based on the affectation of functional motifs within the protein structure, were also analyzed using the domain prediction database Interpro (43) (**Figures 3B–E**). These functional analyses revealed the affectation of an intrinsically disordered region (IDR) in SEMG2 (IDR localization: residues 228–248) (**Figure 3B**). Moreover, Cit was also identified at the prolyl endopeptidase motif (residues 605–635) of the dipeptidyl peptidase IV (DPP-IV) (**Figure 3C**), the peptidase-associated domain of the CBPE protein and the catalytic domain of TGM-4 (**Figures 3D, E**).

## Discussion

The characterization of sEV proteomes from non-NZ men by unbiased discovery-driven proteomics, as performed here, revealed the impaired presence of uPMs in those men affected by infertility. Initially, we encountered increased cumulative signal of hCit residues proteome-wide in sEVs of non-NZ men. An increase in hCit in the human proteome has been associated with unhealthy lifestyle habits such as smoking (44, 45) and systemic clinical syndromes such as atherogenesis or uremia (44, 45). However, no direct associations were identified in this study between the analyzed lifestyle- and clinical-associated variables with the observed accumulation of hCit residues in sEV proteomes. Indeed, our results indicate a negative association between hCit in sEV proteomes of non-NZ men and sperm morphology as well as with progressive motility. Additionally, hCit shows strong abilities to interfere with host immunity (46–48) and balanced immunology is crucial for the entire reproductive process (49). Thus, the tentative hypothesis that an exacerbated presence of hCit in sEVs of non-NZ men may promote abnormal auto-/host-immunity responses affecting the fecundity capa**city** of these subjects deserves further attention.

Citrullination of proteins is catalyzed by PAD enzymes and becomes a calcium-dependent reaction proven to be affected by available calcium levels and PAD sensibility to these ions, as widely reviewed by György et al. (50). Moreover, abnormal calcium metabolism and calcium deficiency have been directly related to apparition and progression of male infertility (51). Thus, whether any of these catalytic factors may affect the lower levels of Cit observed here in key proteins involved in important aspects of male fertility, such as sperm viability, exocytosis, motility, viscosity, inflammation, or the Eppin protein complex, is yet to be deciphered and holds promise to further understand the aberrant occurrence of this uPM in sEVs of non-NZ men. The implications of protein citrullination in crucial physiological processes have also been largely detailed and include gene regulation and protein expression, regulation of molecular signaling, and maintenance of cell and tissue structure [see the excellent review of Maria Christophorou for further detail (52)].

Cit has also been associated with the regulation of protein activation and degradation (53), a fact that may be associated with the differences in the citrullinated/uncitrullinated stoichiometry observed here affecting certain proteins in non-NZ men. Part of the circulating EVs has traditionally been linked to the potential discarding of dysfunctional proteins (12). However, based on the findings encountered in this work, higher unmodified stoichiometry linked to non-NZ sEVs may not support that classical interpretation. It is thus unlikely that sEVs serve degradation and discarding of the noncitrullinated fraction of the proteome, and the presence of lower proteome citrullination stoichiometry in these vesicles may directly contribute to abnormal molecular functions, a hypothesis that, based on the findings observed in this study, needs further scrutinization. Decreased Cit has already been implicated in female infertility and in aberrant embryo development (54, 55). Thus, the abnormal presence of Cit as identified here may involve aberrant signaling of the affected proteins through direct impairment of their expected molecular functions.

The functional *in silico* analyses of protein Cit performed in this study point out that abnormal citrullination in the proteome of non-NZ sEVs does not occur randomly to certain proteins given their physicochemical profiles, but it follows mechanistic rules likely linked to the specific functions of these sEVs molecules. Moreover, Cit affects the proteolytic and catalytic active regions in most of the significantly affected proteins of sEVs in non-NZ men, a fact that may show a high potential to compromise their homeostatic enzymatic activities and requires further research.

Crucial proteins affected by Cit in sEVs of non-NZ play a pivotal role in male fertility, for example, the mitochondrial-associated protein DPP-IV. This protein has been linked to sperm motility and to the induction of premature acrosome reaction when is present in excess on the sperm surface (39, 40). Thus, the abnormal protein Cit profiled observed in the specific subset of proteins have a high chance to mechanistically affect the normal molecular function of these specific proteins in sEVs of non-NZ men, and by chance impact different crucial aspects of the sperm functional quality. Another example that illustrated the importance of Cit in the correct function of protein in male fertility is the affectation of IDRs, unstructured polypeptide segments that do not fold into a defined tertiary structure but still display regulatory and signaling functions (56), by altered Cit profiles. It is known that PTMs modulate the conformational properties and functionality of IDRs (57). Thus, further investigations are required to elucidate the modulating capacity of Cit over the SEMG2 function given its presence within the IDR region.

In a related vein, the potential biomarker ability of the uPM findings encountered here cannot be underestimated and might be considered highly promising, consistent with the report of Candenas et al. (58). The probability of the uPMs reported here to be validated as biomarkers is ~ 20%, based on the Skates et al. calculation power guidelines (59), and requires of specific validation in larger clinical cohorts.

To conclude, our study reports abnormal uPM profiles affecting sEVs of non-NZ men. Specifically, a higher cumulative presence of hCit residues was identified in these seminal plasma proteomes while a specific down-regulated occurrence of protein Cit was found affecting specific proteins and their functional regions in non-NZ men. Furthermore, the aberrant stoichiometry of citrullination was also identified in sEV proteomes of non-NZ subjects. Collectively, these findings identify novel mechanisms linked to aberrant calcium-dependent signaling that may affect the fertility implications of sEVs in sperm. Furthermore, the novel findings reported here pave the way for future studies aimed at identifying the therapeutic potential of aberrant uPMs in sEVs of non-NZ men and to validate their intrinsically promising diagnostic and prognostic biomarker abilities.

## Limitations of the study

Non-NZ subjects did not present a clinical history of exposure to specific gonadotoxin agents (chemotherapy, radiotherapy), testosterone replacement therapy, congenital abnormalities, or severe testicular trauma. However, there are clinical variables such as incipient metabolic syndrome, mild previous testicular trauma, etc. that might be taken into consideration in required further studies aimed to validate the therapeutic target and diagnostic/prognostic capacities of sEVs-uPMs through the use of larger cohorts. Similarly, although NZ subjects did not present any abnormal reproductive clinical history or any clinical sign linked to male infertility, the fact that they have been involved in a previous successful partner pregnancy was not specifically considered an inclusion criterion in this study.

## Data availability statement

All proteomics data and original contributions presented in the study are included in the article/**Supplementary Material**, further inquiries can be directed to the corresponding author/s.

## Ethics statement

The studies involving human participants were reviewed and approved by Ethics Committee of the Autonomous University of Madrid (UAM) with reference: CEI 60-1058. The patients/participants provided their written informed consent to participate in this study.

## Author contributions

RR, AS, and XG-P conceived the idea and designed the experiments. RR and MdlC obtained clinical samples. RR, CL, MM, JM, AB and CE compiled and analyzed the data. AS and XG-P obtained competitive funding, interpreted the data, supervised the project, and wrote the manuscript. All authors contributed to the article and approved the submitted version.

## References

[1] Fainberg J Kashanian JA. Recent advances in understanding and managing male infertility. F1000Res (2019) 8(F1000 Faculty Rev):670. doi: 10.12688/f1000research.17076.1 PMC6524745 31143441

[2] Pereira R Sá R Barros A Sousa M. Major regulatory mechanisms involved in sperm motility. Asian J Androl (2017) 19(1):5–14. doi: 10.4103/1008-682X.167716 26680031 PMC5227674

[3] Waberski D Schäfer J Bölling A Scheld M Henning H Hambruch N. Seminal plasma modulates the immune-cytokine network in the porcine uterine tissue and pre-ovulatory follicles. PloS One (2018) 13(8):e0202654. doi: 10.1371/journal.pone.0202654 30153288 PMC6112639

[4] Roca J Rodriguez-Martinez H Padilla L Lucas X Barranco I. Extracellular vesicles in seminal fluid and effects on male reproduction. an overview in farm animals and pets. Anim Reprod Sci (2021) 246:106853. doi: 10.1016/j.anireprosci.2021.106853 34556398

[5] Simon C Greening DW Bolumar D Balaguer N Salamonsen LA Vilella F. Extracellular vesicles in human reproduction in health and disease. Endocrine Rev (2018) 39(3):292–332. doi: 10.1210/er.2017-00229 29390102

[6] Raposo G Stoorvogel W. Extracellular vesicles: Exosomes, microvesicles, and friends. J Cell Biol (2013) 200(4):373–83. doi: 10.1083/jcb.201211138 PMC3575529 23420871

[7] Gallart-Palau X Serra A Sze SK. Enrichment of extracellular vesicles from tissues of the central nervous system by PROSPR. Mol Neurodegener (2016) 11(1):41. doi: 10.1186/s13024-016-0108-1 27216497 PMC4877958

[8] Lorca C Laparra M Céspedes MV Casaní L Florit S Jové M. Industrial by-products as a novel circular source of biocompatible extracellular vesicles. Adv Funct Mater (2022) 32:2202700. doi: 10.1002/adfm.202202700

[9] Tkach M Théry C. Communication by extracellular vesicles: Where we are and where we need to go. Cell (2016) 164(6):1226–32. doi: 10.1016/j.cell.2016.01.043 26967288

[10] Shetty AK Upadhya R. Extracellular vesicles in health and disease. Aging Dis (2021) 12(6):1358–62. doi: 10.14336/AD.2021.0827 PMC8407881 34527414

[11] Gallart-Palau X Guo X Serra A Sze SK. Alzheimer's disease progression characterized by alterations in the molecular profiles and biogenesis of brain extracellular vesicles. Alzheimers Res Ther (2020) 12(1):54. doi: 10.1186/s13195-020-00623-4 32384937 PMC7210691

[12] Gallart-Palau X Serra A Hase Y Tan CF Chen CP Kalaria RN. Brain-derived and circulating vesicle profiles indicate neurovascular unit dysfunction in early alzheimer's disease. Brain Pathol (2019) 29(5):593–605. doi: 10.1111/bpa.12699 30629763 PMC8028379

[13] Ayaz A Houle E Pilsner JR. Extracellular vesicle cargo of the male reproductive tract and the paternal preconception environment. Syst Biol Reprod Med (2021) 67(2):103–11. doi: 10.1080/19396368.2020.1867665 PMC8272945 33630671

[14] Zhang X Vos HR Tao W Stoorvogel W. Proteomic profiling of two distinct populations of extracellular vesicles isolated from human seminal plasma. Int J Mol Sci (2020) 21(21):7957. doi: 10.3390/ijms21217957 33114768 PMC7663558

[15] Garcia-Rodriguez A de la Casa M Peinado H Gosalvez J Roy R. Human prostasomes from normozoospermic and non-normozoospermic men show a differential protein expression pattern. Andrology (2018) 6(4):585–96. doi: 10.1111/andr.12496 29726126

[16] Janković T Goč S Mitić N Danilović Luković J Janković M. Membrane-associated gamma-glutamyl transferase and alkaline phosphatase in the context of concanavalin a- and wheat germ agglutinin-reactive glycans mark seminal prostasome populations from normozoospermic and oligozoospermic men. Ups J Med Sci (2020) 125(1):10–8. doi: 10.1080/03009734.2019.1690603 PMC7054931 31774341

[17] Mann M Jensen ON. Proteomic analysis of post-translational modifications. Nat Biotechnol (2003) 21(3):255–61. doi: 10.1038/nbt0303-255 12610572

[18] Minguez P Parca L Diella F Mende DR Kumar R Helmer-Citterich M. Deciphering a global network of functionally associated post-translational modifications. Mol Syst Biol (2012) 8(1):599. doi: 10.1038/msb.2012.31 22806145 PMC3421446

[19] Carnino JM Ni K Jin Y. Post-translational modification regulates formation and cargo-loading of extracellular vesicles. Front Immunol (2020) 11:948. doi: 10.3389/fimmu.2020.00948 32528471 PMC7257894

[20] Rosa-Fernandes L Rocha VB Carregari VC Urbani A Palmisano G. A perspective on extracellular vesicles proteomics. Front Chem (2017) 5:102. doi: 10.3389/fchem.2017.00102 29209607 PMC5702361

[21] Bludau I Willems S Zeng W-F Strauss MT Hansen FM Tanzer MC. The structural context of posttranslational modifications at a proteome-wide scale. PloS Biol (2022) 20(5):e3001636. doi: 10.1371/journal.pbio.3001636 35576205 PMC9135334

[22] Gallart-Palau X Serra A Lee BST Guo X Sze SK. Brain ureido degenerative protein modifications are associated with neuroinflammation and proteinopathy in alzheimer's disease with cerebrovascular disease. J Neuroinflammation (2017) 14(1):175. doi: 10.1186/s12974-017-0946-y 28865468 PMC5581431

[23] Turunen S Koivula MK Risteli L Risteli J. Ureido group-specific antibodies are induced in rabbits immunized with citrulline- or homocitrulline-containing antigens. Autoimmunity (2016) 49(7):459–65. doi: 10.3109/08916934.2016.1171853 27098309

[24] Pruijn GJ. Citrullination and carbamylation in the pathophysiology of rheumatoid arthritis. Front Immunol (2015) 6:192. doi: 10.3389/fimmu.2015.00192 25964785 PMC4410602

[25] Turunen S Koivula M-K Nicholas AP Risteli L Risteli J. Homocitrulline: An analog and confounder related to citrulline. In: Nicholas AP Bhattacharya SK, editors. Protein deimination in human health and disease. New York, NY: Springer New York (2014). p. 367–76.

[26] Verheul MK van Veelen PA van Delft MAM de Ru A Janssen GMC Rispens T. Pitfalls in the detection of citrullination and carbamylation. Autoimmun Rev (2018) 17(2):136–41. doi: 10.1016/j.autrev.2017.11.017 29203292

[27] Horibata S Coonrod SA Cherrington BD. Role for peptidylarginine deiminase enzymes in disease and female reproduction. J Reprod Dev (2012) 58(3):274–82. doi: 10.1262/jrd.2011-040 22790870

[28] Barratt CLR Björndahl L De Jonge CJ Lamb DJ Osorio Martini F McLachlan R. The diagnosis of male infertility: An analysis of the evidence to support the development of global WHO guidance-challenges and future research opportunities. Hum Reprod Update (2017) 23(6):660–80. doi: 10.1093/humupd/dmx021 PMC5850791 28981651

[29] Gallart-Palau X Serra A Qian J Chen CP Kalaria RN Sze SK. Temporal lobe proteins implicated in synaptic failure exhibit differential expression and deamidation in vascular dementia. Neurochem Int (2015) 80:87–98. doi: 10.1016/j.neuint.2014.12.002 25497727

[30] Hase Y Polvikoski TM Ihara M Hase M Zafar R Stevenson W. Carotid artery disease in post-stroke survivors and effects of enriched environment on stroke pathology in a mouse model of carotid artery stenosis. Neuropathol Appl Neurobiol (2019) 45(7):681–97. doi: 10.1111/nan.12550 30947376

[31] Serra A Gallart-Palau X Park JE Lim GGY Lim KL Ho HH. Vascular bed molecular profiling by differential systemic decellularization in vivo. Arterioscler Thromb Vasc Biol (2018) 38(10):2396–409. doi: 10.1161/ATVBAHA.118.311552 30354219

[32] Mathivanan S Fahner CJ Reid GE Simpson RJ. ExoCarta 2012: database of exosomal proteins, RNA and lipids. Nucleic Acids Res (2012) 40:D1241–4. doi: 10.1093/nar/gkr828 PMC3245025 21989406

[33] Kalra H Simpson RJ Ji H Aikawa E Altevogt P Askenase P. Vesiclepedia: A compendium for extracellular vesicles with continuous community annotation. PloS Biol (2012) 10(12):e1001450. doi: 10.1371/journal.pbio.1001450 23271954 PMC3525526

[34] Mitchell A Chang HY Daugherty L Fraser M Hunter S Lopez R. The InterPro protein families database: The classification resource after 15 years. Nucleic Acids Res (2015) 43:D213–21. doi: 10.1093/nar/gku1243 PMC4383996 25428371

[35] Zinaman MJ Brown CC Selevan SG Clegg ED. Semen quality and human fertility: A prospective study with healthy couples. J Androl (2000) 21(1):145–53. 10670528

[36] Malenica M Vukomanović M Kurtjak M Masciotti V dal Zilio S Greco S. Perspectives of microscopy methods for morphology characterisation of extracellular vesicles from human biofluids. Biomedicines (2021) 9(6):603. doi: 10.3390/biomedicines9060603 34073297 PMC8228884

[37] Kim DK Kang B Kim OY Choi DS Lee J Kim SR. EVpedia: An integrated database of high-throughput data for systemic analyses of extracellular vesicles. J Extracell Ves (2013) 2. doi: 10.3402/jev.v2i0.20384 PMC3760654 24009897

[38] Keerthikumar S Chisanga D Ariyaratne D Al Saffar H Anand S Zhao K. ExoCarta: A web-based compendium of exosomal cargo. J Mol Biol (2016) 428(4):688–92. doi: 10.1016/j.jmb.2015.09.019 PMC4783248 26434508

[39] Nath D Shaw M. Characterization of a dipeptidyl peptidase and its role in motility of rat epididymal maturing spermatozoa. Reprod Biol Insights (2015) 8:9. doi: 10.4137/RBI.S34737

[40] Pérez-Patiño C Li J Barranco I Martínez EA Rodriguez-Martínez H Roca J. The proteome of frozen-thawed pig spermatozoa is dependent on the ejaculate fraction source. Sci Rep (2019) 9(1):705. doi: 10.1038/s41598-018-36624-5 30679492 PMC6345957

[41] Yamasaki K Yoshida K Yoshiike M Shimada K Nishiyama H Takamizawa S. Relationship between semenogelins bound to human sperm and other semen parameters and pregnancy outcomes. Basic Clin Androl (2017) 27:15. doi: 10.1186/s12610-017-0059-6 28794880 PMC5547539

[42] Samanta L Sharma R Cui Z Agarwal A. Proteomic analysis reveals dysregulated cell signaling in ejaculated spermatozoa from infertile men. Asian J Androl (2019) 21(2):121–30. doi: 10.4103/aja.aja_56_18 PMC6413549 30381577

[43] Hunter S Apweiler R Attwood TK Bairoch A Bateman A Binns D. InterPro: The integrative protein signature database. Nucleic Acids Res (2009) 37:211–5. doi: 10.1093/nar/gkn785 PMC2686546 18940856

[44] Wang H Huang B Wang W Li J Chen Y Flynn T. High urea induces depression and LTP impairment through mTOR signalling suppression caused by carbamylation. eBioMedicine (2019) 48:478–90. doi: 10.1016/j.ebiom.2019.09.049 PMC6838447 31628020

[45] Berg AH Drechsler C Wenger J Buccafusca R Hod T Kalim S. Carbamylation of serum albumin as a risk factor for mortality in patients with kidney failure. Sci Transl Med (2013) 5(175):29. doi: 10.1126/scitranslmed.3005218 PMC3697767 23467560

[46] Shi J Knevel R Suwannalai P MPvd L Janssen GMC Veelen P. Autoantibodies recognizing carbamylated proteins are present in sera of patients with rheumatoid arthritis and predict joint damage. Proc Natl Acad Sci USA (2011) 108(42):17372–7. doi: 10.1073/pnas.1114465108 PMC3198314 21987802

[47] Turunen S Hannonen P Koivula M-K Risteli L Risteli J. Separate and overlapping specificities in rheumatoid arthritis antibodies binding to citrulline- and homocitrulline-containing peptides related to type I and II collagen telopeptides. Arthritis Res Ther (2015) 17(1):2. doi: 10.1186/s13075-014-0515-z 25573503 PMC4320812

[48] Cook KW Xue W Symonds P Daniels I Gijon M Boocock D. Homocitrullination of lysine residues mediated by myeloid-derived suppressor cells in the tumor environment is a target for cancer immunotherapy. J Immunother Cancer (2021) 9(7):e001910. doi: 10.1136/jitc-2020-001910 34321274 PMC8320257

[49] Mohamed Khosroshahi L Parhizkar F Kachalaki S Aghebati-Maleki A Aghebati-Maleki L. Immune checkpoints and reproductive immunology: Pioneers in the future therapy of infertility related disorders? Int Immunopharmacol (2021) 99:107935. doi: 10.1016/j.intimp.2021.107935 34304000

[50] György B Tóth E Tarcsa E Falus A Buzás EI. Citrullination: A posttranslational modification in health and disease. Int J Biochem Cell Biol (2006) 38(10):1662–77. doi: 10.1016/j.biocel.2006.03.008 16730216

[51] Beigi Harchegani A Irandoost A Mirnamniha M Rahmani H Tahmasbpour E Shahriary A. Possible mechanisms for the effects of calcium deficiency on Male infertility. Int J Fertil Steril (2019) 12(4):267–72. doi: 10.22074/ijfs.2019.5420 PMC6186280 30291684

[52] Christophorou MA. The virtues and vices of protein citrullination. R Soc Open Sci (2022) 9(6):220125. doi: 10.1098/rsos.220125 35706669 PMC9174705

[53] Qu Y Olsen JR Yuan X Cheng PF Levesque MP Brokstad KA. Small molecule promotes β-catenin citrullination and inhibits wnt signaling in cancer. Nat Chem Biol (2018) 14(1):94–101. doi: 10.1038/nchembio.2510 29083417

[54] Christophorou MA Castelo-Branco G Halley-Stott RP Oliveira CS Loos R Radzisheuskaya A. Citrullination regulates pluripotency and histone H1 binding to chromatin. Nature (2014) 507(7490):104–8. doi: 10.1038/nature12942 PMC4843970 24463520

[55] Xu Y Shi Y Fu J Yu M Feng R Sang Q. Mutations in PADI6 cause female infertility characterized by early embryonic arrest. Am J Hum Genet (2016) 99(3):744–52. doi: 10.1016/j.ajhg.2016.06.024 PMC5010645 27545678

[56] Babu MM. The contribution of intrinsically disordered regions to protein function, cellular complexity, and human disease. Biochem Soc Trans (2016) 44(5):1185–200. doi: 10.1042/BST20160172 PMC5095923 27911701

[57] Bah A Forman-Kay JD. Modulation of intrinsically disordered protein function by post-translational modifications. J Biol Chem (2016) 291(13):6696–705. doi: 10.1074/jbc.R115.695056 PMC4807257 26851279

[58] Candenas L Chianese R. Exosome composition and seminal plasma proteome: A promising source of biomarkers of Male infertility. Int J Mol Sci (2020) 21(19):7022. doi: 10.3390/ijms21197022 32987677 PMC7583765

[59] Skates SJ Gillette MA LaBaer J Carr SA Anderson L Liebler DC. Statistical design for biospecimen cohort size in proteomics-based biomarker discovery and verification studies. J Proteome Res (2013) 12(12):5383–94. doi: 10.1021/pr400132j PMC4039197 24063748

