# Supplementary material for: Altered ureido protein modification profiles in seminal plasma extracellular vesicles of non-normozoospermic men
Source: Front Endocrinol (Lausanne). 2023 Mar 22;14:1113824. doi: 10.3389/fendo.2023.1113824 (PMC10073716; doi:10.3389/fendo.2023.1113824)
Supplement: Supplementary file 1 [file Table_1.docx]

**Supplementary Material**

**Altered ureido protein modifications profiles in seminal plasma extracellular vesicles of non-normozoospermic men**

Rosa Roy, Cristina Lorca, María Mulet, Jose Antonio Sánchez Milán, Alejandro Baratas, Moisés de la Casa, Carme Espinet, Aida Serra^†,*^and Xavier Gallart-Palau^†,*^

^†^ A.S. and X.G.-P. as joint senior authors. * Corresponding authors.

**Summary table:**

| **Content** | **Page** |
| --- | --- |
| **Supplementary Figure S1.** Linear correlation between protein concentration and particles concentration in sEVs samples | S-2 |
| **Supplementary Figure S2.** Total intensity of citrullinated (Cit) proteins in sEVs proteomes detected in Non-NZ and NZ. | S-3 |
| **Supplementary Table S1.** Demographic, clinical and lifestyle data from NZ and non-NZ donors. | S-4 |
| **Supplementary Table S2.** Characterization of isolated sEVs. | S-6 |
| **Supplementary Dataset S1:** List of citrullinated peptides (Cit) identified by iTraq-quantitative liquid chromatography tandem mass spectrometry in sEVs from NZ (NZ 1-3) and non-NZ (non-NZ 1-5). | Additional file |
| **Supplementary Dataset S2:** List of carbamylated peptides identified by iTraq-quantitative liquid chromatography tandem mass spectrometry in sEVs from NZ (NZ 1-3) and non-NZ (non-NZ 1-5). | Additional file |

^
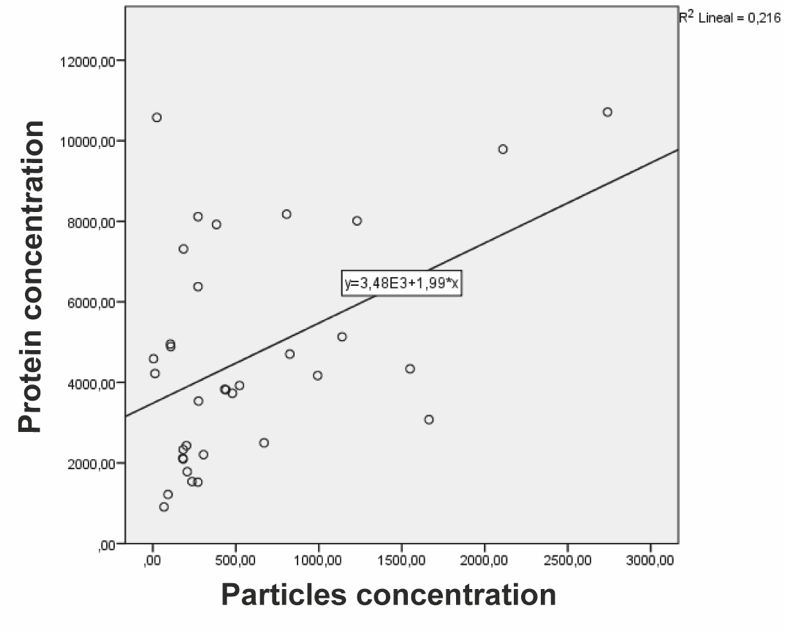
^

**Supplementary Figure S1.** Linear correlation between protein concentration and particles concentration in sEVs samples (Spearman´s Rho= 0.337, P=0.051).

^
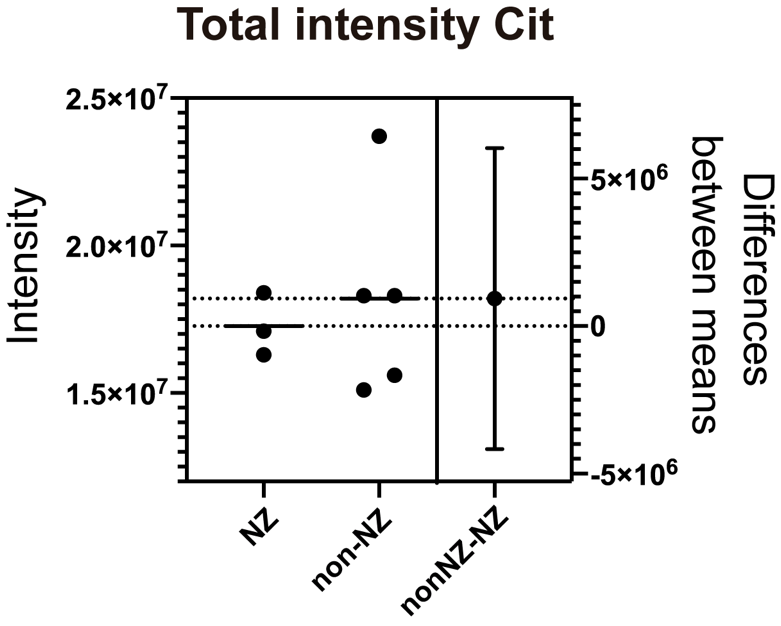
^

**Supplementary Figure S2.** Total intensity of citrullinated (Cit) proteins in sEVs proteomes detected in non-NZ and NZ. Difference between Non-NZ and NZ (NoNon-NZZ-NZ) is displayed using the right Y-axis. Intensity was calculated as sum of spectral count of all Cit-modified peptides detected.

**Supplementary Table S1.** Demographic, clinical and lifestyle data from NZ and non-NZ donors. ^a^Smoking habit expressed as cigarettes/day. ^b^ N/D refers to not determined. ^c^CDT refers to Crohn's disease treatment. ^d^FN refers to 5-alpha reductase inhibitor finasteride.

| **Subject** | **Smoking^a^** | **Alcohol consumption** | **Age** | **Toxicology** | **Allergies** | **Medication** | **Atherogenicity** | **Urology**  **history** | **Previous offspring** | **Clinical sperm parameters** | | | | |
| --- | --- | --- | --- | --- | --- | --- | --- | --- | --- | --- | --- | --- | --- | --- |
|  |  |  |  |  |  |  |  |  |  | **Volume (mL)** | **Count (mill/mL)** | **Progressive Motility (%)** | **Morphology**  **(%)** | **Vitality**  **(%)** |
| NZ1 | No | No | 38 | No | N/D | Statins/Alopurinol | Yes | No | No | 3 | 45 | 60 | 10 | 80 |
| NZ2 | No | No | 40 | No | N/D | No | NO | No | Yes | 4 | 120 | 80 | 8 | 69 |
| NZ3 | N/D^b^ | N/D | N/D | N/D | N/D | N/D | N/D | N/D | N/D | 5 | 58 | 50 | 5 | 75 |
| NZ4 | No | No | 43 | No | No | No | No | No | Yes | 3 | 53 | 60 | 6 | 70 |
| NZ5 | No | No | 42 | No | No | Statins | Yes | No | No | 3.4 | 41 | 40 | 4 | 78 |
| NZ6 | 2-3 | No | 38 | No | No | No | No | No | No | 4 | 46 | 35 | 4 | 80 |
| NZ7 | 15 | No | 41 | Yes | Yes | No | No | No | No | 1.5 | 43 | 60 | 6 | 85 |
| NZ8 | No | No | 37 | No | Pollen | No | No | No | No | 6 | 26 | 50 | 5 | 85 |
| NZ9 | 10 | No | 23 | No | No | No | No | No | No | 4.2 | 42 | 65 | 5 | 80 |
| NZ10 | 6 | No | 33 | No | No | No | No | No | No | 4 | 28 | 50 | 4 | 83 |
| NZ11 | 3 | No | 26 | Cannabis | No | No | No | No | No | 2.4 | 23 | 35 | 6 | 60 |
| NZ12 | No | No | 22 | No | No | No | No | No | No | 2.4 | 92 | 60 | 12 | 85 |
| Non-NZ1 | No | No | 41 | No | No | No | No | No | No | 2 | 36 | 45 | 3 | 80 |
| Non-NZ2 | No | No | 37 | N/D | No | CDT^c^ | No | No | No | 4 | 35 | 40 | 3 | 80 |
| Non-NZ3 | 10 | No | 39 | No | No | No | No | No | No | 4 | 45 | 60 | 3 | 92 |
| Non-NZ4 | No | No | 40 | No | No | No | No | No | Yes | 3.2 | 16 | 40 | 2 | 73 |
| Non-NZ5 | No | Low | 45 | No | No | No | No | No | Yes | 3.5 | 34 | 30 | 4 | 66 |
| Non-NZ6 | No | No | 42 | No | N/D | N/D | N/D | N/D | N/D | 4 | 23 | 30 | 4 | 66 |
| Non-NZ7 | No | No | 40 | No | Ibuprofen | No | No | No | No | 3.5 | 17 | 30 | 3 | 68 |
| Non-NZ8 | No | No | 34 | No | No | No | No | No | No | 2.5 | 32 | 25 | 3 | 66 |
| Non-NZ9 | 4 | No | 46 | No | Pollen/mites | No | No | No | No | 3.5 | 41 | 25 | 3 | 72 |
| Non-NZ10 | No | No | 51 | No | No | NO | No | No | No | 4.9 | 23 | 30 | 3 | 66 |
| Non-NZ11 | 8 | No | 37 | No | No | FN^d^ | No | Hydrocele | No | 3 | 3.5 | 30 | 3 | 65 |
| Non-NZ12 | 10 | No | 43 | No | No | Statins | Yes | No | No | 0.5 | 1.2 | 20 | 2 | 66 |
| Non-NZ13 | No | No | 36 | No | No | N/D | N/D | No | No | 3 | 12 | 30 | 2 | 69 |
| Non-NZ14 | N/D | N/D | N/D | N/D | N/D | N/D | N/D | N/D | N/D | 2.1 | 11 | 20 | 3 | 63 |

**Supplementary Table S2.** Interaction analysis of age with relevant clinical signs that show significant difference in non-NZ compared to NZ. Interaction significance was assessed by Pearson R correlation (*p* < 0.05).

| **Variables** | **Pearson R** | | | **95% confidence interval** |
| --- | --- | --- | --- | --- |
|  | **P value** | **R square** | **r** |  |
| Age vs. Motility | 0.9349 | 0.00031 | -0.0176 | -0.4180 to 0.3886 |
| Age vs. Morphology | 0.8993 | 0.000745 | -0.0273 | -0.4260 to 0.3803 |
| Age vs. Vitality | 0.3823 | 0.03487 | -0.1867 | -0.5488 to 0.2343 |
| Age vs. Count | 0.2977 | 0.04917 | 0.2217 | -0.1995 to 0.5738 |

**Supplementary Table S3.** Characterization of isolated sEVs.

| **Sample** | **Subject** | **Particle concentration**  **(x10^+12^/mL)** | **Medium diameter**  **(nm)** | **Protein**  **(µg/mL)** |
| --- | --- | --- | --- | --- |
| **1** | NZ1 | 10.6 | 116 | 4953 |
|  | NZ2 | 18.5 | 113 | 7313 |
|  | NZ3 | 4.00 | 126 | 4586 |
|  | NZ4 | 2.07 | 135 | 1783 |
| **2** | NZ5 | 1.08 | 163 | 4888 |
|  | NZ6 | 3.83 | 155 | 7922 |
|  | NZ7 | 1.83 | 134 | 2097 |
|  | NZ8 | 4.33 | 168 | 3831 |
| **3** | NZ9 | 2.36 | 183 | 1538 |
|  | NZ10 | 1.83 | 158 | 2333 |
|  | NZ11 | 16.64 | 152 | 3076 |
|  | NZ12 | 11.40 | 125 | 5133 |
| **4** | Non-NZ1 | 4.40 | 177 | 3815 |
|  | Non -NZ2 | 2.02 | 187 | 2430 |
|  | Non -NZ3 | 8.06 | 147 | 8176 |
|  | Non -NZ4 | 13.00 | 135 | 4220 |
| **5** | Non -NZ5 | 1.80 | 161 | 2116 |
|  | Non -NZ6 | 9.93 | 127 | 4170 |
| **6** | Non -NZ7 | 2.72 | 166 | 6378 |
|  | Non -NZ8 | 2.75 | 148 | 3537 |
|  | Non -NZ9 | 12.30 | 171 | 8012 |
|  | Non -NZ10 | 21.10 | 120 | 9789 |
| **7** | Non -NZ11 | 0.91 | 159 | 1217 |
|  | Non -NZ12 | 0.67 | 184 | 908 |
|  | Non -NZ13 | 2.72 | 71 | 8116 |
|  | Non -NZ14 | 8.25 | 125 | 4703 |
